# Increasing Temperature and Relative Humidity Accelerates Inactivation of SARS-CoV-2 on Surfaces

**DOI:** 10.1128/mSphere.00441-20

**Published:** 2020-07-01

**Authors:** Jennifer Biryukov, Jeremy A. Boydston, Rebecca A. Dunning, John J. Yeager, Stewart Wood, Amy L. Reese, Allison Ferris, David Miller, Wade Weaver, Nathalie E. Zeitouni, Aaron Phillips, Denise Freeburger, Idris Hooper, Shanna Ratnesar-Shumate, Jason Yolitz, Melissa Krause, Gregory Williams, David G. Dawson, Artemas Herzog, Paul Dabisch, Victoria Wahl, Michael C. Hevey, Louis A. Altamura

**Affiliations:** a National Biodefense Analysis and Countermeasures Center (NBACC), Operated by Battelle National Biodefense Institute (BNBI) for the U.S. Department of Homeland Security Science and Technology Directorate, Fort Detrick, Maryland, USA; b Censeo Consulting Group Inc., Washington, DC, USA; University of Maryland School of Medicine

**Keywords:** COVID-19, SARS-CoV-2, contamination, coronavirus, fomite, half-life, humidity, temperature, transmission

## Abstract

Coronavirus disease 2019 (COVID-19) was first identified in China in late 2019 and is caused by newly identified severe acute respiratory syndrome coronavirus 2 (SARS-CoV-2). Previous studies had reported the stability of SARS-CoV-2 in cell culture media and deposited onto surfaces under a limited set of environmental conditions. Here, we broadly investigated the effects of relative humidity, temperature, and droplet size on the stability of SARS-CoV-2 in a simulated clinically relevant matrix dried on nonporous surfaces. The results show that SARS-CoV-2 decayed more rapidly when either humidity or temperature was increased but that droplet volume (1 to 50 μl) and surface type (stainless steel, plastic, or nitrile glove) did not significantly impact decay rate. At room temperature (24°C), virus half-life ranged from 6.3 to 18.6 h depending on the relative humidity but was reduced to 1.0 to 8.9 h when the temperature was increased to 35°C. These findings suggest that a potential for fomite transmission may persist for hours to days in indoor environments and have implications for assessment of the risk posed by surface contamination in indoor environments.

**IMPORTANCE** Mitigating the transmission of SARS-CoV-2 in clinical settings and public spaces is critically important to reduce the number of COVID-19 cases while effective vaccines and therapeutics are under development. SARS-CoV-2 transmission is thought to primarily occur through direct person-to-person transfer of infectious respiratory droplets or through aerosol-generating medical procedures. However, contact with contaminated surfaces may also play a significant role. In this context, understanding the factors contributing to SARS-CoV-2 persistence on surfaces will enable a more accurate estimation of the risk of contact transmission and inform mitigation strategies. To this end, we have developed a simple mathematical model that can be used to estimate virus decay on nonporous surfaces under a range of conditions and which may be utilized operationally to identify indoor environments in which the virus is most persistent.

## Abstract (precis)

Mitigating the transmission of SARS-CoV-2 in clinical settings and public spaces is critically important to reduce the number of COVID-19 cases while effective vaccines and therapeutics are under development. SARS-CoV-2 transmission is thought to primarily occur through direct person-to-person transfer of infectious respiratory droplets or through aerosol-generating medical procedures. However, contact with contaminated surfaces may also play a significant role. In this context, understanding the factors contributing to SARS-CoV-2 persistence on surfaces will enable a more accurate estimation of the risk of contact transmission and inform mitigation strategies. To this end, we have developed a simple mathematical model that can be used to estimate virus decay on nonporous surfaces under a range of conditions and which may be utilized operationally to identify indoor environments in which the virus is most persistent.

## OBSERVATION

Several studies have reported the presence of severe acute respiratory syndrome coronavirus 2 (SARS-CoV-2) genetic material on surfaces in coronavirus disease 2019 (COVID-19) patient rooms and throughout hospital wards, indicating environmental contamination. Commonly contaminated items included office equipment (e.g., mice, keyboards, printers) and metal furnishings (e.g., doorknobs, handrails) (1–5). However, those studies did not evaluate the presence of infectious virus on surfaces in a clinical setting. Disposable exam gloves also represent a source of fomite transmission, and this risk is likely elevated when they are used by individuals unaccustomed to proper use, doffing, and disposal of personal protective equipment (PPE).

Two previous laboratory studies demonstrated persistence of infectious SARS-CoV-2 on numerous surfaces over extended periods; however, each of these studies evaluated only a single set of indoor environmental conditions and used virus suspended in cell culture media. Using 50 μl of virus-containing droplets deposited onto nonporous (copper, stainless steel, and plastic) and porous (cardboard) surfaces at 21 to 23°C and 40% relative humidity (RH), van Doremalen et al. were able to show persistence of virus on plastic and stainless steel for up to 72 h, whereas the durations of persistence on cardboard (24 h) and copper (4 h) were much shorter (6). Chin et al. evaluated the stability of 5-μl droplets of SARS-CoV-2 deposited on several porous (paper, wood, cloth, banknote, face mask) and nonporous (glass, stainless steel, and plastic) surfaces at 22°C and 65% RH. Infectious virus was detected on nonporous surfaces for 2 to 4 days, whereas stability on porous surfaces lasted 30 min to 2 days (7). Although these initial studies provided valuable insights into the potential for fomite transmission in a variety of contexts, it is difficult to fully interpret and generalize these results. First, the previous studies did not assess the efficiency of virus recovery from surfaces, and so infectious decay cannot be separated from potential physical losses. Also, it is unclear what degree of precision was achieved in maintaining temperature and RH in the prior studies. More broadly, viral persistence under indoor conditions is complex and may be driven by many factors, including surface type, temperature, relative humidity (RH), and matrix (e.g., bodily fluids) (8–12). Here, we report the first analysis of the stability of SARS-CoV-2 in simulated saliva, to represent a relevant clinical matrix (13, 14), using droplets of various sizes deposited on surfaces and incubated under a range of controlled temperature and RH conditions, thereby providing a more complete understanding of factors that influence SARS-CoV-2 environmental persistence.

**FIG 1:**
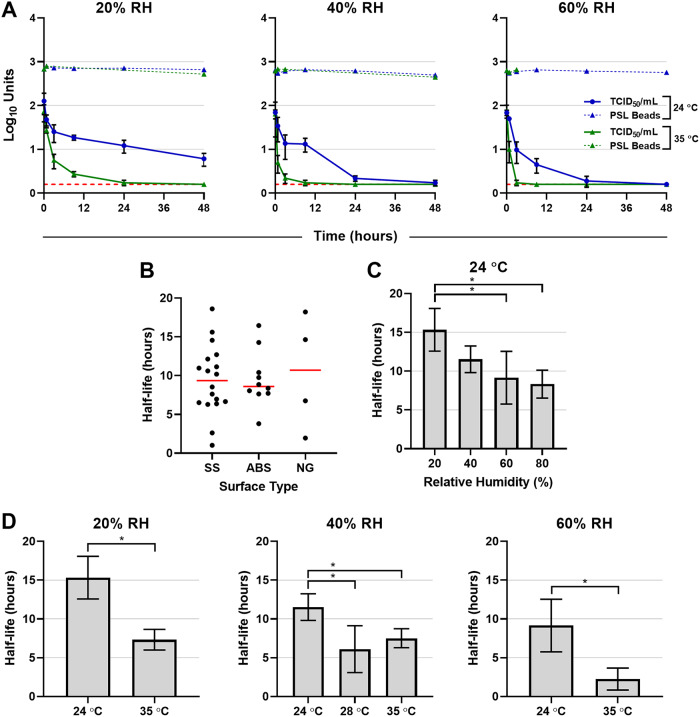
Impact of droplet size, surface type, temperature, and relative humidity on SARS-CoV-2 decay. SARS-CoV-2 or fluorescently labeled polystyrene latex (PSL) beads were diluted 1:10 in simulated saliva, and then droplets (1, 5, or 50 μl) were deposited onto stainless steel (SS), acrylonitrile butadiene styrene (ABS) plastic, or nitrile glove (NG) coupons and incubated within an environmentally controlled chamber. During each experiment, three virus test coupons and one PSL bead coupon were chosen randomly at various time points over a period of 48 h. Both virus and PSL beads were recovered by resuspension into culture medium and then either quantified by a cell-based infectivity assay on Vero cells to determine the median tissue culture infectious dose (TCID_50_/ml) or assayed for fluorescence with a multimode plate reader. The mean half-life estimates (measured in hours) for SARS-CoV-2 in simulated saliva under each set of conditions were derived from fitting a generalized linear model with a normal distribution and identity link function to infectivity data from each trial over time. (A) Representative data comparing SARS-CoV-2 infectivity (TCID_50_/ml) and PSL microsphere relative fluorescence units (RFU) across a range of temperature and RH conditions. Data from six of the trials are shown where 5-μl droplets were deposited onto stainless steel. Red horizontal dashed lines indicate the lower limit of detection for the TCID_50_ assay (0.2 log_10_ TCID_50_/ml). Error bars indicate standard deviations for each time point. (B) Estimated virus half-life on different surfaces from all 32 trials across a range of a droplet sizes, temperatures, and relative humidities. Individual half-life estimates are shown as points, and the mean half-life value for each surface is indicated by a horizontal line. (C and D) Comparisons of virus half-life estimates among various RH (20 to 80%) conditions (C) and temperature (24 and 35°C) and RH (20 to 80%) conditions (D). Bars represent means of results from each group, and error bars indicate standard deviations. Statistical significance (*P* < 0.05) is indicated by brackets and asterisks (*). A one-way ANOVA and a Student’s *t* test were used to calculate the data presented in panels C and D, respectively.

To determine the stability of SARS-CoV-2 on surfaces, virus was diluted 1:10 in simulated saliva and droplets were deposited onto stainless steel, acrylonitrile butadiene styrene (ABS) plastic, or nitrile rubber glove coupons. These surfaces were chosen to represent two common sources of fomite transmission (door knobs/handles and office electronics) and also to address the risks associated with contaminated PPE. Virus stability was measured using multiple RH and temperature combinations ranging from approximately 20 to 80% RH and 24 to 35°C. Coupons were placed in an environmentally controlled testing plenum, which maintained RH and temperature set points with low variability over a total of 32 trials. During each trial, virus was recovered from three randomized coupons into culture medium at predetermined time points over a period of 48 h. The amount of infectious virus remaining was determined using a quantitative cell-based infection assay. Virus infectivity data collected over time were fitted to statistical models to estimate the infectivity decay rate and half-life (*t*_1/2_) for each set of experimental conditions. A single coupon loaded with 0.1-μm-diameter fluorescently labeled polystyrene latex microspheres was also collected at each time point, resuspended in buffer, and then evaluated for fluorescence. These controls showed that there was not a significant decrease in fluorescence over time (Fig. 1A), and the data suggest that there was no significant physical loss but that the reduction in virus titers represented biological decay. In a subset of the trials (*n* = 6), we also quantitated viral RNA using reverse transcription quantitative real-time PCR (RT-qPCR) as a second measure of physical losses but observed that its decay rate was dependent on both temperature and humidity conditions and thus concluded that viral RNA could not be used as a physical tracer (data not shown). Although this observation requires further experimental validation, these data suggest that the utility of viral RNA for contamination surveillance may be confounded by environmental conditions.

Using virus deposited onto stainless steel coupons at ambient indoor temperature (24°C) and maintained using various levels of RH (20, 40, 60, and 80%), we found that droplet size was not a significant factor influencing the half-life of SARS-CoV-2 (one-way analysis of variance [ANOVA], *P* = 0.39; Bartlett’s test, *P* = 0.28); as a result, we did not further evaluate droplet size on ABS plastic or nitrile rubber. Comparison of levels of virus decay determined from all 32 trials, which used a variety of volumes, temperatures (24, 28, and 35°C), and RH conditions, showed there was not a significant difference in half-life estimates between virus deposited on stainless steel, ABS plastic, or nitrile glove coupons (one-way ANOVA, *P* = 0.93; Bartlett’s test, *P* = 0.26) (Fig. 1B). Based on these comparisons, data from all trials were combined for subsequent analyses.

We estimated the mean half-life values in hours (± standard deviations [SD]) for RH of 20% (*t*_1/2_ = 15.33 ± 2.75), 40% (*t*_1/2_ = 11.52 ± 1.72), 60% (*t*_1/2_ = 9.15 ± 3.39), and 80% (*t*_1/2_ = 8.33 ± 1.80) at 24°C, with the results indicating that virus at ambient indoor temperature is most stable at relatively low RH (Fig. 1C). Pairwise comparisons of RH conditions at 24°C performed using the Tukey-Kramer method found the half-life at 20% RH to be significantly different from the half-life at both 60 and 80% RH (*P* = 0.0038 and *P* = 0.0023, respectively). We also evaluated the effect of temperature on the surface stability of SARS-CoV-2 (Fig. 1D). When temperature was increased from 24 to 35°C, the higher temperature resulted in faster virus decay and shorter half-life at 20% RH (*t*_1/2_ = 7.33 ± 1.33), 40% RH (*t*_1/2_ = 7.52 ± 1.22), and 60% RH (*t*_1/2_ = 2.26 ± 1.42). Pairwise comparisons of mean half-life results between 24 and 35°C under each set of RH conditions (Student’s *t* test) demonstrated that these differences were significant (*P* = 0.0016, 0.0327, and 0.0170, respectively). At 40% RH, virus decay was also evaluated at 28°C (*t*_1/2_ = 6.11 ± 3.02) and found to be significantly faster (Student’s *t* test; *P* = 0.0163). The combination of 35°C and 80% RH could not be evaluated due to test system limitations.

**FIG 2:**
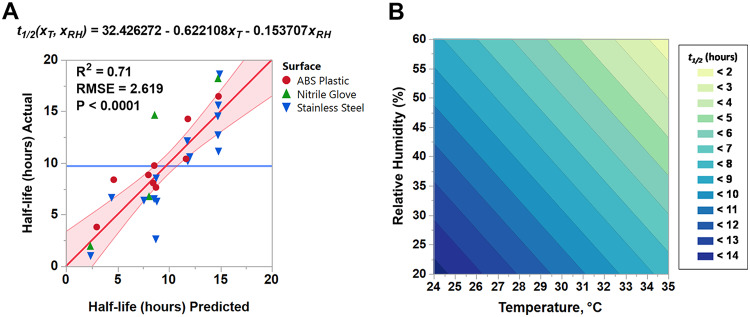
Temperature and humidity response model for SARS-CoV-2 decay. (A) Using empirically determined estimates of SARS-CoV-2 half-life data collected under various temperature and RH conditions (24 to 35°C, 20 to 60% RH), a regression analysis was performed to determine a predictive decay model. The equation shown, where *x_T_* is temperature (°C) and *x_RH_* is percent RH, can be used to estimate half-life (*t*_1/2_) (in hours) for SARS-CoV-2 on stainless steel, ABS plastic, or nitrile glove rubber within the range of environmental conditions tested in this study. Actual half-life data are plotted against predicted half-life estimates. The mean of all data is indicated by a horizontal blue line. The regression fit line is shown in red. The shaded region indicates the 95% confidence interval of the regression fit line. RMSE, root mean square error. (B) Contour plot representing the predicted estimates of SARS-CoV-2 half-life data in simulated saliva on stainless steel, ABS plastic, or nitrile rubber as a function of temperature and RH.

Analysis of estimated half-life data from 28 trials by two-way ANOVA demonstrated that both temperature and RH were significant factors influencing virus decay (*P* < 0.0001 for both). Furthermore, we were able to fit a linear regression equation (adjusted *R*^2^ = 0.71) that models the half-life of SARS-CoV-2 in simulated saliva on nonporous surfaces (stainless steel, ABS plastic, and nitrile rubber) (Fig. 2A). This model can also be represented as a contour plot and used to estimate mean viral persistence at any combination of temperature and RH conditions within the design space (i.e., 20 to 60% RH and 24 to 35°C) (Fig. 2B). Trials performed at the 80% RH condition (*n* = 4) were not included in the model, as we were able to evaluate 80% RH only at 24°C. Although our data indicate that the virus half-life was inversely proportional to increasing temperature and RH, extrapolated estimates for conditions beyond those empirically tested may not be valid.

These data provide the first evidence that increasing temperature and/or RH decreased how long SARS-CoV-2 remained infectious on two hard, nonporous surfaces representative of commonly contaminated objects, suggesting that persistence and, ultimately, exposure risk may vary significantly depending on environmental conditions. In addition, our data on virus stability on nitrile gloves serve as a reminder that PPE, when used inappropriately, has the potential to be a source of contamination.

Although indoor temperatures can be effectively controlled by HVAC (heating, ventilation, and air conditioning) systems, in the absence of active humidity control, indoor RH is influenced by outdoor weather conditions. The data shown here are most relevant for indoor conditions as they do not address the significant effect of solar radiation upon virus decay (15). However, these data could be applied to virus decay on surfaces under outdoor conditions at night when environmental conditions are within the range tested in this study. These data can inform disinfection protocols for hospital rooms or mobile COVID-19 treatment facilities, as environments with relatively low humidity may warrant more frequent cleaning. Temporarily increasing temperature and/or RH in these spaces once they are vacated may also complement cleaning with chemical disinfectants to reduce the risk of potential fomite transmission.

While we have demonstrated the impact of RH and temperature on the stability of SARS-CoV-2 on stainless steel and ABS plastic as representative nonporous surfaces, the applicability of these results to porous surfaces (e.g., clothing, masks, cardboard packaging) is currently unknown and bears further investigation. In addition, data regarding the amount of infectious virus present on surfaces in patient rooms are required in order to determine how long such surfaces would remain a source of virus transmission. Finally, the infectious dose of SARS-CoV-2 is currently unknown and thus it is difficult to evaluate the risk of infection following contact with a contaminated fomite. Despite these caveats, these data can be used to determine how long it would take to reduce levels of infectious virus on nonporous surfaces by a specific factor (e.g., 99.9% reduction after 10 × *t_1/2_*) under a wider range of environmental conditions than was previously possible.

### Cell culture.

Vero (ATCC CCL-81) cells were used to propagate SARS-CoV-2 and in virus microtitration assays. Cells were cultured at 37°C and 5% CO_2_ in complete growth medium (gMEM) as previously described (16). A ViaFill reagent dispenser (Integra Biosciences Corp.) was utilized to seed cells into 96-well, clear-bottom plates for virus microtitration assays. Cells were seeded at a density appropriate to achieve approximately confluent monolayers on the day of infection.

### Virus stock production.

SARS-CoV-2 isolate USA-WA1/2020 (NR-52281) was deposited by the Centers for Disease Control and Prevention and obtained through BEI Resources, NIAID, NIH. This virus (passage 4) was propagated twice in Vero cells to yield the working stocks (passage 6) that were used for this work. Briefly, a volume of 2.4 × 10^6^ Vero cells suspended in 30 ml of gMEM was combined with SARS-CoV-2 at a multiplicity of infection equal to 0.01 and was then seeded into a T-150 flask and incubated at 37°C and 5% CO_2_. After 24 h, a suspension of 1.2 × 10^6^ uninfected Vero cells mixed in 0.5 ml gMEM was added to the infected cells. At 72 h postinfection, the flasks were frozen at –80°C for at least 1 h and then thawed at 37°C, and a cell scraper was used to remove any remaining cells from the flask. The combination of the infected cell lysate and culture supernatant was clarified by centrifugation at 1,000 × *g* for 10 min at 4°C. Virus was aliquoted and stored at –80°C prior to use in experiments. The viral stocks were sequenced and found to match the consensus sequence previously described (MN985325.1). All work with SARS-CoV-2 was performed under conditions of biosafety level 3 containment.

### Test matrix.

SARS-CoV-2 was diluted 1:10 in simulated saliva to approximate the behavior of virus in a relevant bodily fluid. Simulated saliva was prepared according to previous recipes (17, 18) (Table 1), with the exceptions of KH_2_PO_4_ and K_2_HPO_4_, which were present at 15.4 mM and 24.6 mM, respectively. The simulated saliva was characterized for its pH, surface tension, viscosity, percent solids, and protein content (Table 2) and found to be similar to that described in previous reports (17, 18). The pH was measured using a SevenExcellence pH meter (Mettler-Toledo). Surface tension was measured at settings of both 15 and 750 ms on a SITA pro line t15 bubble pressure tensiometer (SITA Process Solutions). Viscosity was measured using a SV-1A tuning fork vibro viscometer (A&D Company, Ltd.). Percentages of solids were analyzed using a MA35 infrared moisture analyzer (Sartorius). To quantify protein content, a Pierce bicinchoninic acid (BCA) protein assay kit (Thermo Fisher Scientific; catalog no. 23225) was used with an albumin standard, and tests were read on a SpectraMax M5 plate reader (Molecular Devices). Simulated saliva was stored at 4°C for up to 2 weeks prior to use in experiments.

**TABLE 1:** Composition of simulated saliva

Composition	Concn
MgCl_2_ · 6 H_2_O	196.75 μM
CaCl_2_ · H_2_O	1.17 mM
NaHCO_3_	5.00 mM
KH_2_PO_4_	15.4 mM
K_2_HPO_4_	24.6 mM
NH_4_Cl	2.06 mM
KSCN	1.96 mM
(NH_2_)_2_CO	2.00 mM
NaCl	15.06 mM
KCl	13.95 mM
Mucin^*a*^	0.3% (wt/vol)
Distilled water	To volume

a Mucin, porcine gastric mucin type III (Millipore Sigma).

**TABLE 2:** Characterization of simulated saliva

Characteristic	Values
Protein concn	0.68 ± 0.00 mg/ml
pH	7.3 ± 0.0
Viscosity	0.88 ± 0.00 Pa·s × 10^−3^
Surface tension (15 ms)	72.87 ± 0.24 mN/m
Surface tension (750 ms)	71.77 ± 0.33 mN/m
% solids	1.01 ± 0.10

### Surface decay test system.

To determine the biological decay rate (i.e., loss of infectivity) of SARS-CoV-2 on stainless steel or plastic, virus-laden wet droplets were deposited onto coupons, which were then incubated under various RH and temperature conditions, followed by recovery of virus from the coupons for microtitration infectivity assays. For each test performed on either grade 304 stainless steel (Diamond Perforated Metals) or ABS plastic, sterilized 19-mm-diameter circular coupons were loaded with 1, 5, or 50 μl of SARS-CoV-2 diluted 1:10 in simulated saliva. For each test performed on nitrile gloves, a 0.25-in. square cut from nitrile gloves was placed on a stainless steel coupon and loaded with 5 μl of SARS-CoV-2 diluted 1:10 in simulated saliva. The coupons were immediately placed into a custom 20-port temperature- and relative humidity-controlled plenum supplied with constant airflow at 5 liters/min. Humidity control was performed by mixing streams of HEPA-filtered dry and humidified air with mass flow controllers (Alicat Scientific). Humid air was generated by circulating ultrapure water through Nafion gas humidification bundles (Perma Pure). For temperatures in the range of 24°C, the plenum was equilibrated to room temperature. For temperatures outside this range, the plenum temperature was controlled by circulating a sealed loop of chilled or heated water around the plenum. Temperature and RH were monitored continuously via temperature/RH probes inserted directly into the plenum (Vaisala), and data were collected with either LabVIEW (National Instruments) or Vaisala Insight PC software. During the experiments, temperature fluctuated ±2°C and RH (20, 40, 60, or 80%) was controlled at ±10%. In each experiment, the initial virus titer was determined by immediately placing virus-laden coupons into 4 ml gMEM, vortex mixing for 30 s at 2,400 rpm, and then quantifying infectivity via microtitration assay as described below. To control for physical efficiency and sample extraction, 0.1-μm-diameter green fluorescent polystyrene latex microspheres (Thermo Fisher Scientific; catalog no. G100B) diluted 1:1 in gMEM were spotted onto coupons (1, 5, or 50 μl), incubated in the test plenum with the virus-laden coupons, and recovered as described for the virus, and fluorescence (expressed in relative fluorescence units [RFU]) was measured on a GloMax luminometer (Promega). Viral RNA was quantified (as described below) in a subset of the samples. All coupons were processed identically at 0.75-, 3-, 9-, 24-, and 48-h time points.

### Virus microtitration assay.

Virus containing samples were serially diluted (10^−1^ through 10^−4^) in 96-well, clear-bottom plates containing confluent monolayers of Vero cells. For each dilution, a total of 10 replicate wells were infected. The infected plates were incubated at 37°C and 5% CO_2_ for 4 days, and then individual wells were visually inspected using a Nikon TS100 microscope for the presence of virus-induced cytopathic effects (CPE) at each dilution compared to a negative (medium only) control. The median 50% tissue culture infectious dose (TCID_50_) was estimated for each of the samples using the Spearman-Karber method (19, 20).

### Viral nucleic acid quantification.

SARS-CoV-2 RNA was quantified by a reverse transcription quantitative real-time PCR (RT-qPCR) assay using an Applied Biosystems 7500 Fast real-time PCR instrument and SuperScript III one-step RT-PCR MasterMix with Platinum *Taq* DNA polymerase. The target of the PCR assay is a conserved region of the viral RNA-dependent RNA polymerase (RdRp) gene. Briefly, viral RNA from test samples was isolated and purified using the Qiagen Viral RNA Mini Kit centrifugation protocol per the manufacturer’s instructions. The PCR master mix was composed of sterile, molecular biology-grade water, 1× SuperScript reaction mix, 1× SuperScript reverse transcriptase, 0.2 μM forward and reverse primers, and 0.1 μM 6-carboxyfluorescein (FAM)-labeled fluorescent probe. The primers and probe were based on primer and probe sequences published previously by Corman et al. (21) but were modified to replace the redundant bases with consensus bases. Reaction plates were set up by combining 5 μl of RNA and 15 μl of PCR master mix per well. Each plate also had a 7-point standard curve based on a synthetic DNA positive control representing the assay’s target amplicon (gBlocks; Integrated DNA Technologies). Cycling conditions were run as follows: hold for 50°C for 30 min, 95°C for 10 min, and then 40 cycles of 95°C for 15 s and 60°C for 1 min. Quantification was determined by the number of cycles required to cross a threshold of 0.02 (values reported as threshold cycles [*C_T_*]). Data representing numbers of viral RNA copies present in test samples per milliliter were interpolated from the standard curves.

### Design of experiments.

Trials performed on both stainless steel and ABS plastic encompassed a complete design space that included 5-μl droplets incubated at 24°C (20, 40, 60, and 80% RH), 28°C (40% RH), and 35°C (20, 40, and 60% RH). On stainless steel, additional trials were performed using 1-μl and 50-μl droplets at 24°C (20, 40, 60, and 80% RH). For analyses using nitrile glove rubber, a reduced design space was utilized that permitted comparison between surface types, and trials were performed at 24°C (20 and 60% RH) and 35°C (20 and 60% RH).

### Statistical analysis.

ANOVA was used to compare the effects of droplet size and surface on the decay half-life. Bartlett’s test was used to show that there was no statistical difference in half-life variance across the droplet size or surface type data. Analysis of the data indicated that virus decay was biphasic and was related to the drying time of virus-containing droplets. Thus, each data set was divided into a wet phase and a dry phase. For 20, 40, and 60% RH, the wet phase was defined from time point 0 to 0.75 h and the dry phase was from time point 0.75 h to 48 h. For 80% RH, the wet phase was defined from time point 0 to 3 h and the dry phase was from time point 3 h to 48 h. These estimates were based on observations of droplet drying during experiments as well as on RH data collected from the surface test decay system (data not shown).

A logarithm (base 10) transformation was applied to the TCID_50_ data, and a linear model was fitted using MATLAB’s FITGLM with an identify link function and a normal distribution. Points that were below the assay’s limit of detection (0.2 log_10_ TCID_50_/ml) were removed from the analysis. The half-life (*t*_1/2_) was also determined as follows:

$$t_{1/2}=\frac{{\text{log}}_{10}2}{k}$$

Regression analysis was applied to determine the dependence of the virus half-life on temperature and relative humidity. The following model was initially assumed where *x_T_* represents temperature in degrees Celsius, *x*_RH_ represents percent relative humidity, and the β terms represent coefficients:

$$t_{1/2}\left(x_{T},x_{\text{RH}}\right)={\text{β}}_{0}+{\text{β}}_{1}x_{T}+{\text{β}}_{2}x_{\text{RH}}+{\text{β}}_{3}x_{T}x_{\text{RH}}+{\text{β}}_{4}x_{T}^{2}+{\text{β}}_{5}x_{\text{RH}}^{2}$$

Stepwise regression (MATLAB STEPWISELM.M) was then used to identify and remove predictors that were insignificant using a backward elimination approach. The stepwise procedure starts with the full model and then measures the contribution of each predictor to the residual sum of squared errors (SSE) using an *F*-test on the ratio of the SSE with and without that predictor; the most statistically insignificant predictor (if there is one) is then removed, and the process is repeated until no further predictors can be removed without a statistically significant loss in the SSE. After determining the model, an *F*-test was performed to confirm that the model provided a better fit than an intercept-only model. The fitted residuals were then analyzed to verify the absence of any apparent structure, and the Shapiro-Wilke test was used to verify residual normality. The final model for the decay of SARS-CoV-2 on stainless steel, ABS plastic, and nitrile rubber as a function of temperature in degrees Celsius and percent relative humidity is as follows:

$$t_{1/2}\left(x_{T},x_{\text{RH}}\right)=32.426272-0.622108x_{T}-0.153707x_{\text{RH}}$$

It should be noted that this model is valid for the range of conditions tested; extrapolation outside the range of temperature and relative humidity values tested is not advised and should be done with caution.

### Data visualization.

GraphPad Prism version 8.4 was used to generate graphs. JMP Genomics version 9.1 was used to prepare a contour plot of half-life versus temperature and RH.
